# Liposome-Embedding Silicon Microparticle for Oxaliplatin Delivery in Tumor Chemotherapy

**DOI:** 10.3390/pharmaceutics12060559

**Published:** 2020-06-17

**Authors:** Armando Cevenini, Christian Celia, Stefania Orrù, Daniela Sarnataro, Maddalena Raia, Valentina Mollo, Marcello Locatelli, Esther Imperlini, Nicoletta Peluso, Rosa Peltrini, Enrica De Rosa, Alessandro Parodi, Luigi Del Vecchio, Luisa Di Marzio, Massimo Fresta, Paolo Antonio Netti, Haifa Shen, Xuewu Liu, Ennio Tasciotti, Francesco Salvatore

**Affiliations:** 1 Dipartimento di Medicina Molecolare e Biotecnologie Mediche, Università degli Studi di Napoli “Federico II”, 80131 Napoli, Italy; armando.cevenini@unina.it (A.C.); sarnatar@unina.it (D.S.); nicoletta.peluso@email.it (N.P.); rossella.peltrini@gmail.com (R.P.); luigi.delvecchio@unina.it (L.D.V.); 2 CEINGE-Biotecnologie Avanzate S.c.a r.l., 80145 Napoli, Italy; orru@uniparthenope.it (S.O.); raia@ceinge.unina.it (M.R.); 3 Department of Pharmacy, University of Chieti—Pescara “G. d’Annuzio”, 66100 Chieti, Italy; c.celia@unich.it (C.C.); m.locatelli@unich.it (M.L.); luisa.dimarzio@unich.it (L.D.M.); 4 Department of Nanomedicine, Houston Methodist Research Institute, Houston, TX 77030, USA; ederosa@houstonmethodist.org (E.D.R.); hshen@houstonmethodist.org (H.S.); xliu@houstonmethodist.org (X.L.); 5 Dipartimento di Scienze Motorie e del Benessere, Università “Parthenope”, 80133 Napoli, Italy; 6 IRCCS SDN, 80143 Napoli, Italy; imperlini@ceinge.unina.it (E.I.); aparodi.sechenovuniversity@gmail.com (A.P.); 7 Italian Institute of Technology@CRIB Center for Advanced Biomaterials for Health Care, 80125 Napoli, Italy; valentina.mollo@iit.it (V.M.); paoloantonio.netti@unina.it (P.A.N.); 8 Department of Respiratory Sciences, College of Life Sciences, University of Leicester, Leicester LE1 7RH, UK; 9 Institute of Molecular Medicine, Sechenov First Moscow State Medical University, 119991 Moscow, Russia; 10 Department of Health Sciences, University “Magna Græcia” of Catanzaro, Campus Universitario “S. Venuta”, I-88100 Catanzaro, Italy; fresta@unicz.it; 11 Department of Chemical, Materials & Industrial Production Engineering, University of Naples Federico II, 80125 Naples, Italy; 12 Department of Cell and Developmental Biology, Weill Cornell Medicine, New York, NY 10065, USA; 13 Center for Biomimetic Medicine, Houston Methodist Research Institute (HMRI), Houston, TX 77030, USA; 14 Houston Methodist Orthopedics & Sports Medicine, Houston Methodist Hospital, Houston, TX 77030, USA

**Keywords:** mesoporous silicon microparticle, nanoparticle, liposome, multistage vector, oxaliplatin, colon cancer

## Abstract

Mesoporous silicon microparticles (MSMPs) can incorporate drug-carrying nanoparticles (NPs) into their pores. An NP-loaded MSMP is a multistage vector (MSV) that forms a Matryoshka-like structure that protects the therapeutic cargo from degradation and prevents its dilution in the circulation during delivery to tumor cells. We developed an MSV constituted by 1 µm discoidal MSMPs embedded with PEGylated liposomes containing oxaliplatin (oxa) which is a therapeutic agent for colorectal cancer (CRC). To obtain extra-small liposomes able to fit the 60 nm pores of MSMP, we tested several liposomal formulations, and identified two optimal compositions, with a prevalence of the rigid lipid 1,2-distearoyl-sn-glycero-3-phosphocholine and of 1,2-distearoyl-sn-glycero-3-phosphoethanolamine-N-[methoxy(polyethylene glycol)-2000]. To improve the MSV assembly, we optimized the liposome-loading inside the MSMP and achieved a five-fold increase of the payload using an innovative lyophilization approach. This procedure also increased the load and limited dimensional changes of the liposomes released from the MSV in vitro. Lastly, we found that the cytotoxic efficacy of oxa-loaded liposomes and-oxa-liposome-MSV in CRC cell culture was similar to that of free oxa. This study increases knowledge about extra-small liposomes and their loading into porous materials and provides useful hints about alternative strategies for designing drug-encapsulating NPs.

## 1. Introduction

The delivery of intravenously injectable therapeutic nanoparticles (NPs) into tumors is hampered by several biologic barriers. Consequently, the amount of therapeutics accumulating in tumor tissues is limited thereby resulting in low antitumor efficacy and high adverse side effects [1,2]. The first biologic barriers encountered by NPs after injection are the hemodynamic barrier and clearance of the mononuclear phagocytic system (MPS) [3,4]. A PEG coating on NPs can help to overcome MPS clearance [3] and can significantly prolong circulation in the bloodstream [4], the hemodynamic barrier can physically prevent NPs efficiently marginating towards the endothelial walls. In this context, it was shown that, thanks to the erythrocyte flux, a 1–3 µm mesoporous silicon microparticle (MSMP) tends to circulate close to the endothelial walls in the space designated the “cell-free layer” [5] and that it can accommodate, within its pores, a variety of NPs carrying therapeutic molecules [6].

The shape of the MSMPs was conceived via mathematical modeling to: (i) circulate in the bloodstream; (ii) efficiently marginate towards the endothelial walls of the blood vessels; (iii) accumulate in the vessels where the circulation is not efficient, like tumor capillary [7,8,9], which also have a fenestrated endothelium. There, the MSMPs can adhere to the endothelial walls and release their therapeutic payload through a constant degradation of their silicon structure. Hence the released nanotherapeutics can pass through the endothelium fenestrae thereby exploiting the enhanced permeability and retention (EPR) effect and finally reaching the tumor [10].

The MSMP loaded with NPs constitutes a multistage vector (MSV) with three stages of compartmentalization (MSMP, NP and therapeutic molecules) that form a Matryoshka-like structure. The MSMP protects its cargo from degradation and accumulate in tumor vascular endothelium [6,11,12] thereby releasing *on site* the NPs which are able to cross the endothelial walls [3,4,6,13]. Thus, the composite vector preserves the biopharmaceutical properties of payloads, limits the effect of the hemodynamic barrier and facilitates the transport and the confinement of therapeutics to pathologic tissues [6,11,12,13,14,15,16,17,18]. In fact, the effectiveness of the MSV in vivo has been shown in several reports [6,11,13,18,19,20,21,22].

Among the NPs that can be used as constituent of the MSV, PEGylated liposomes are particularly effective in cancer therapy. We previously demonstrated that such MSV improved the delivery of paclitaxel-loaded liposomes to bone marrow [23]. This type of platform also enhanced the effect of injection of anti-EphA2 siRNA-loaded liposomes against ovarian tumors [24]. In fact, the MSMP enables the transport and release of small liposomes in tumor tissues where they accumulate according to the EPR effect [25,26].

Among the various MSMPs tested for drug delivery as the first stage of a MSV, a discoidal MSMP measuring 1 µm in diameter and 400-nm-thick proved to be very effective in transporting different payloads inside the silicon pores of its internal structure. The MSVs based on this type of MSMP also proved to be very effective in delivering the payloads to a variety of tumors in animal models [6,7,11,12,27,28,29,30]. The aim of the present study was to use this type of MSMP, coupled to PEGylated liposomes, to develop a MSV able to deliver therapeutic agents for colorectal cancer (CRC).

Colorectal cancer is one of the leading causes of cancer mortality [31]. Indeed, there was an estimated 101,420 new cases and 51,020 deaths in 2019 in the USA [32]. Therapeutic protocols for CRC include oxaliplatin (oxa) in combination with 5-fluorouracil (5-FU) and folinic acid (FOLFOX) or capecitabine (XELOX). The composition of the therapeutic cocktail and treatment duration can vary depending on CRC stage and clinical parameters of the patients [33]. Oxa is relatively better tolerated than other platinum derivatives, but still elicits adverse events mainly gastrointestinal toxicity, hematologic side effects and neurotoxicity [34]. In fact, oxa causes a peripheral sensory neuropathy depending on the oxalate ligand of the drug that has a direct effect on the voltage-gated Na^+^ and K^+^ channels and an indirect effect on Ca^2+^ and Mg^2+^ channels [35].

To overcome some of these drawbacks, we synthesized oxa-containing small PEGylated liposomes, under 50 nm in diameter, which were then loaded inside the pores of the MSMP to obtain an assembled MSV therapeutic system. The entrapment of the oxa-loaded small PEGylated liposomes in the MSV may protect against the toxicity of the platinum-based drug, increase oxa stability and safeguard oxa pharmaceutical properties without altering its therapeutic efficacy versus CRC cells. Indeed, our aim was to obtain an MSV carrying small preformed lipidic NPs with a narrow size distribution. To achieve this goal, we challenged the physical limits of liposome assembly to produce extra-small lipidic NPs able to fit the 60-nm pores of the MSMP, testing several liposomal formulations and eventually identifying two optimal lipidic compositions.

To overcome a foreseeable payload limit related to the extra-small cavity of the newly synthetized liposomes, we devised an innovative method with which to load a large amount of liposomes into the MSMP pores by introducing a lyophilization step in the protocol. Oxa-liposomes and oxa-liposome-MSVs were physicochemically characterized (size, polydispersion index, ζ-potential), and then their cytostatic efficacy was evaluated in vitro in a CRC cell line and in primary vascular endothelial cells.

## 2. Materials and Methods

### 2.1. Materials

L-α-phosphatidylcholine (PC) (from chicken egg), 1,2-dipalmitoyl-sn-glycero-3-phosphoethanolamine (PE), 1,2-distearoyl-sn-glycero-3-phosphoethanolamine-N-[methoxy(polyethylene glycol)-2000] (DSPEmPEG2000), 1,2-dioleoyl-sn-glycero-3-phosphocholine (DOPC), 1,2-dipalmitoyl-sn-glycero-3-phosphocholine (DPPC) and 1,2-distearoyl-sn-glycero-3-phosphocholine (DSPC) were purchased from Avanti Polar Lipids, Inc. (Alabaster, AL, USA). Oregon Green-conjugated 1,2-dihexadecanoyl-sn-glycero-3-phosphoethanolamine (DHPE), Alexa Fluor 546-conjugated phalloidin, 4’,6-diamidino-2-phenylindole dihydrochloride (DAPI) and ProLong™ Gold antifade mountant were purchased from Thermo Fisher Scientific (Waltham, MA, USA). Porous polycarbonate membranes (Whatman^®^ Nuclepore track-etched membranes) for extrusion, 4-(2-hydroxyethyl)piperazine-1-ethanesulfonic acid (HEPES), dialysis tubes with a 12 kDa cut-off, glutaraldehyde, phosphate buffered saline (PBS) pH 7.4 (137 mM NaCl, 2.7 mM KCl, 8 mM Na_2_HPO_4_ and 2-mM KH_2_PO_4_.), sodium cacodylate, osmium tetroxide, oxaliplatin (oxa), carboplatin, L-glutamine, penicillin/streptomycin solution, McCoy 5A medium, trypsin/EDTA solution, 3-(4,5-dimethyl-2-thiazolyl)-2,5-diphenyl-2H-tetrazolium bromide (MTT), hydrogen chloride, trypan blue solution, hydrogen peroxide, isopropyl alcohol (IPA), 3-(aminopropyl)-triethoxysilane (APTES) (IUPAC name: 3-triethoxysilylpropan-1-amine), porcine skin gelatin and bovine serum albumin (BSA) were purchased from Sigma-Aldrich (St. Louis, MO, USA). Chloroform, methanol (HPLC-grade), acetonitrile (HPLC-grade), sulfuric acid were purchased from Carlo Erba (Milan, Italy). MilliQ water was produced using a Millipore Milli-Q Plus water management system (Millipore Bedford Corp., Bedford, MA, USA). EBM-2 medium, EGM-2 kit and fetal bovine serum (FBS) were purchased from Lonza, Ltd. (Basel, Switzerland). Poly/Bed^®^ Epoxy resin was purchased from Polysciences, Inc. (Warrington, PA, USA). 8-well Falcon™ Chambered cell culture slides were purchased from Corning Life Sciences (Tewksbury, MA, USA). 4% (*w/v*) paraformaldehyde in PBS was purchased from Santa Cruz Biotechnology (Dallas, TX, USA).

### 2.2. Equipment

Liposome production: rotating evaporator Büchi Rotavapor R-210 (Büchi, Milan, Italy); Lipex Extruder^TM^ (Northern Lipids, Inc., Burnaby, BC, Canada); Hielscher UP200H probe sonicator (Hielscher, Teltow, Germany). Liposome physicochemical characterization by dynamic light scattering (DLS) and laser doppler electrophoresis (LDE): Malvern Zetasizer Nano ZS90 (Malvern Instrument, Ltd., Malvern, UK). Transmission electron microscopy (TEM) and cryo-TEM: TECNAI 20 G2 electron microscope equipped with an Eagle 2HS camera, Vitrobot Mark IV plunge freezer, (FEI, Thermo Fisher Scientific, Electron Solution Microscopy, Hillsboro, OR, USA); Leica Ultramicrotome, Leica EM stainer (Leica, Deerfield, IL, USA). HPLC analyses: HPLC Thermo Fisher Scientific liquid chromatography (model Spectra System P2000) equipped with a diode array detector (DAD, mod. Spectra System UV6000LP); Spectra System SCM1000 degasser (Thermo Fisher Scientific, Waltham, MA, USA); Zorbax Extended C-18 packing column (250 × 4.6 mm, 5 μm particle size); security guard column (4.0 × 3.0 mm, 5-μm particle size); Jetstream2 Plus column oven; Excalibur v.2.0 Software (Thermo Fisher Scientific, Waltham, MA, USA). Spectrophotometry and spectrofluorimetry: PerkinElmer EnSpire microplate reader (PerkinElmer, Waltham, MA, USA); GraphPad Prism software (GraphPad Software, San Diego, CA, USA). Cytofluorimetry: BD FACSCanto II and BD FACSDiva™ software (BD Biosciences, Franklin Lakes, NJ, USA). Drying: SpeedVac (Savant) Savant™ SpeedVac™ concentrator (Thermo Fisher Scientific, Waltham, MA, USA). Lyophilization: Edwards Modulyo lyophilizer (Edwards Lifesciences, Irvine, CA, USA). Confocal microscopy: Leica TCS SP5 confocal microscope equipped with a 40× oil-immersion objective, Leica “LAS AF” software (Leica Biosystems, Wetzlar, Germany).

### 2.3. MSMP Production

Mesoporous silicon microparticles (MSMPs) were generated as previously reported [11,12,36,37] by photolithography and electrochemical etching. The MSMPs used in the present study have a discoidal shape with 1-μm diameter, 0.4-μm thickness and 60-nm (mean diameter) pores [11,12].

### 2.4. Liposome Production

Liposomes were produced by using different lipid combinations (Table 1). Liposomes were obtained using the thin layer evaporation method, as described elsewhere [38]. Briefly, 40 mg of lipids were dissolved in chloroform/methanol (CH_3_OH/CHCl_3_) (1: 3 *v*/*v*), then the organic solvent was removed by evaporation in a rotating evaporator Büchi Rotavapor R-210 at 90 rpm, at the mean (of the lipid mixture) transition temperature (Tm), for 30 min. The lipid film was hydrated with 2 mL of buffer 10-mM HEPES pH 7.4, with or without (empty liposomes) 2.5 mg/mL oxa, with three cycles, each consisting of 3 min heating at the Tm indicated in Table 1, and 3 min of vortex-mixing at 25 rpm. The vesicle suspension was then extruded with a Lipex Extruder^TM^ (Northern Lipids, Inc.) by using porous polycarbonate membranes with a progressive reduction of pore sizes (600, 200, 100, 50 nm) followed by sonication or by further extrusion with 30 nm pores. Sonication was performed at the mean Tm of phospholipids with a probe sonicator (Hielscher, model UP200H) equipped with an exponential microprobe operating at 24 kHz and amplitude of 60%, in order to obtain unilamellar suspensions.

Liposomal formulations were dialyzed in 10-mM HEPES by using dialysis tubes with a 12-kDa cutoff (Sigma-Aldrich) to remove non-encapsulated oxa. Fluorescent liposomes were obtained by including 0.5% (*w/w*) Oregon Green-conjugated DHPE (Thermo Fisher Scientific) in the lipid composition during the preparation of lipid film.

### 2.5. Physicochemical Characterization of Liposomes

Hydrodynamic diameter (Dh), the polydispersity index (PDI) and the ζ-potential of liposomes were analyzed as previously reported [39,40] using a Malvern Zetasizer Nano ZS90 (Malvern Instrument, Ltd.). For both analyses, liposome suspensions were diluted to 100 µg/mL in 10-mM HEPES pH 7.4 to avoid multiscattering phenomena. Dh and PDI were measured by dynamic light scattering (DLS), using as a light source the 4.5-mW laser diode of the Zetasizer and using a scattering angle of 173°. The third order cumulants method was applied as fitting of the autocorrelation function. The following parameters were used during the analysis: real refractive index (1.59), an imaginary refractive index (0.0), a medium refractive index (1.330), a medium viscosity (1.0 mPa·s) and medium dielectric constant (80.4).

The ζ-potential was measured by laser Doppler electrophoresis (LDE) using the 5.0 mW He/Ne laser (633 nm) of the Zetasizer Nano ZS90. A Smoluchowski constant F (Ka) of 1.5 was used during the analysis.

### 2.6. Transmission Electron Microscopy (TEM)

For Cryo- TEM analysis of oxa-loaded liposomes, 3 µL of liposome suspension (20 µg lipids/µL in 10-mM HEPES pH 7.4) were spread over a copper grid (200 mesh with carbon membrane). After removing excess solution, the grid was plunged into liquid propane and then transferred into a box in liquid nitrogen in a Vitrobot Mark IV plunge freezer (FEI, Thermo Fisher Scientific, Electron Solution Microscopy). Afterward, the grid was put into the cryo-holder which was then transferred into a TECNAI 20 G2 (FEI, Thermo Fisher Scientific, Electron Solution Microscopy) microscope equipped with an Eagle 2HS camera and the images were acquired under low electron–dose conditions (~5–20 electrons/Å).

For TEM analysis of MSV, human umbilical vein endothelial cells (HUVEC) (Lonza, cod. C2517A) were seeded in 10 cm plates at a density of 2 × 10^6^ cells/cm^2^. Twenty-four hours later, the cells were incubated for 3 h with 200 MSV (or empty MSMP) particles/cell. Subsequently, the cells were washed twice with PBS and fixed in a solution of glutaraldehyde 2.5% (*v*/*v*) (Sigma-Aldrich) and 0.1-M sodium cacodylate (Sigma-Aldrich) for 2 h at room temperature. After three washes with sodium cacodylate 0.1 M, the cells were post-fixed with 1% (*v*/*v*) osmium tetroxide for 1 h and then washed twice for 5 min in 0.1-M sodium cacodylate. The cells were dehydrated in increasing concentrations of ethanol (30%, 50%, 70%, 95%, 99%) and then embedded in Poly/Bed^®^ Epoxy resin. The samples were polymerized in an oven at 60 °C for 2 days and ultrathin sections (50 nm) were cut in a Leica Ultramicrotome, stained with uranyl acetate and lead citrate in a Leica EM Stainer, and examined in a TECNAI 20 G2 microscope at an accelerating voltage of 80 kV.

### 2.7. Oxaliplatin Encapsulation Efficiency

Liposomal formulations were dialyzed in 10-mM HEPES by using dialysis tubes with a 12 KDa cutoff (Sigma-Aldrich) to remove unentrapped oxa. Liposome suspension was then dried in a Savant SpeedVac concentrator (Thermo Fisher Scientific), dissolved in methanol and loaded onto an HPLC (Spectra System P2000, Thermo Fisher Scientific) equipped with a C18 column. Samples were analyzed using the HPLC method (see Materials and Methods, Section 2.8). Empty liposomes served as controls during the analysis.

### 2.8. HPLC Analysis and Validation

The HPLC analysis and validation method of oxa-loaded liposomes were carried out as previously reported [41]. Briefly, oxa and carboplatin (Internal Standard, IS) were dissolved in MilliQ water and a stock solution of oxa was obtained. The standard solution was further diluted to obtain working standards for HPLC analysis. Stock solution, working solutions, calibration curve, sample preparation and method validation were carried out according to the protocols included in Supplementary Materials (Section S2.1) and were in agreement with previous works [42] and international guidelines for validation analysis [43].

The analysis was carried out using an HPLC Thermo Fisher Scientific liquid chromatography (model Spectra System P2000) equipped with a diode array detector (DAD, mod. Spectra System UV6000LP) and an online Spectra System SCM1000 degasser (Thermo Fisher Scientific). The data analysis was performed with the Excalibur v.2.0 Software (Thermo Fisher Scientific). Oxa and IS were resolved and quantified using a Zorbax Extended C-18 packing column (250 × 4.6 mm, 5-μm particle size) coupled to a security guard column (4.0 × 3.0 mm, 5-μm particle size). A Jetstream2 Plus column oven was used to thermostate the column at 25 °C (±1 °C) during the analysis. The initial mobile phase was a solution of MilliQ water and acetonitrile (98:2, *v:v*) at a flow rate of 1.0 mL/min. Oxa and IS were separated by a gradient elution and were detected at the maximum wavelength of 249 (Supplementary Materials, Table S1 and Figure S1).

### 2.9. Cell Cultures

Primary human umbilical vein endothelial cells (HUVEC) (Lonza, cod. C2517A) were cultured in EBM-2 (Lonza) medium supplemented with EGM-2 kit (Lonza). Colon cancer HCT-116 (ATCC, cod. CCL-247) cells were cultured in McCoy 5A medium (Sigma-Aldrich) supplemented with 10% (*v*/*v*) fetal bovine serum (FBS) (Lonza), 2-mM L-glutamine (Sigma-Aldrich), 1% (*v*/*v*) penicillin/streptomycin solution (Sigma-Aldrich). Cell cultures were maintained in an incubator at 37 °C and in humidified atmosphere with 5% CO_2_.

### 2.10. MTT Assay

Cell viability was evaluated by 3-(4,5-dimethyl-2-thiazolyl)-2,5-diphenyl-2H-tetrazolium bromide (MTT) assay as reported elsewhere [44]. Briefly, 3 × 10^3^ HUVEC or 1.2 × 10^4^ HCT-116 cells were seeded in each well of 96-well plates (Corning). After 24 h, cells were washed with PBS and the culture medium was replaced with fresh medium containing proper concentrations of liposomes (0.001; 0.002; 0.01; 0.02; 0.1; 0.2 µg lipids/mL) or MSVs (0.1; 1.0; 5.0; 10.0; 50 × 10^4^ particle/mL) or oxaliplatin (0.05; 0.25; 0.5; 2.5; 5; 25 µM) (free or encapsulated). After 24, 48 and 72 h of incubation with liposomes, MSV or oxaliplatin, cells were washed with PBS and the culture medium was replaced with fresh medium containing 0.5 mg/mL of MTT (Sigma-Aldrich). The cells were incubated for 2 h at 37 °C and then washed with PBS. Then, the cells were incubated for 2 h at 37 °C in culture medium containing 0.5 mg/mL of MTT (Sigma-Aldrich) and subsequently washed with PBS. After PBS removal, a solution of 1-N hydrogen chloride-isopropanol (1:24, *v*/*v*) was added to each well and the plate was kept under shaking at room temperature for few minutes. Finally, the absorbance at 570 nm of the samples was measured using a PerkinElmer EnSpire microplate reader. The absorbance values were normalized versus untreated cells (control) and expressed as cell viability%. Experiments were performed in five independent replicates. IC_50_ was calculated by nonlinear regression [log(inhibitor) vs. response—variable slope (four parameters)] of cell viability data, using GraphPad Prism software. The one-way two-tailed paired *t-*test was used to calculate statistical significance (*p*-value). Differences were considered significant at *p* < 0.05.

### 2.11. Cellular Uptake of Liposomes

3 × 10^4^ cells/cm^2^ of HUVEC and 9 × 10^4^ cells/cm^2^ of HCT-116 were seeded in 6-well plates. After 24 h, cells were washed with PBS, and the culture medium was replaced with fresh medium containing 0.2 mg/mL of fluorescent liposomes. After 3 h of incubation, cells were washed with PBS, detached by trypsin/EDTA solution (Sigma-Aldrich), resuspended with PBS and centrifuged at 230× *g* for 10 min at 4 °C. Then the supernatant was discarded, and cell pellets were resuspended in PBS containing 1 mg/mL of trypan blue, to quench extracellular fluorescence due to possible not internalized liposomes [45]. Cells were analyzed by cytofluorimetry, using a BD FACSCanto II and BD FACSDiva™ software (BD Biosciences). The mean fluorescence intensity (MFI) values of control cells (non-incubated with liposomes), due to cell autofluorescence, were considered “background” and were subtracted from the MFI of liposome-treated cells. The values were then normalized with the corresponding fluorescent intensity of liposome suspensions (0.2 mg lipids/mL, in PBS), measured by spectrofluorimetry with a PerkinElmer EnSpire microplate reader. Thereafter the normalized values were divided by 10^3^ to optimize the format for graphical representation. The experiments were performed in three independent replicates and averages and standard deviations of normalized values were reported as arbitrary units (A.U.).

### 2.12. Surface Charge Modification of MSMP

MSMPs were oxidized using the piranha etching protocol consisting of incubation in sulfuric acid and hydrogen peroxide (3:1, *v*/*v*) as previously described [46,47]. Oxidized MSMPs were stored at 4 °C in isopropyl alcohol (IPA) and used within two months. Oxidized MSMPs were modified with 3-(aminopropyl)-triethoxysilane (APTES) (Sigma-Aldrich) as previously reported [48]. Briefly, 1 × 10^9^ of oxidized MSMPs were dried under vacuum in a SpeedVac (Savant) for 8 h. Dried particles were incubated in 0.5 mL of a solution 2% APTES, 4.9% IPA, 93.1% H_2_O 2% (*v*/*v*/*v*) for 2 h, at 35 °C, under shaking at 1300 rpm. Particles were then centrifuged at 4000× *g* for 10 min, and after the supernatant was discarded, they were dried under vacuum in a SpeedVac (Savant) for 8 h. APTES-modified MSMP were stored at 4 °C in isopropyl alcohol (IPA) and used within two weeks.

### 2.13. MSV Assembly

For the MSV assembly by incubation, the loading of liposomes into the APTES-modified MSMP was performed using a fluorescent-liposome suspension at a concentration of 20 mg lipids/mL in 10-mM HEPES pH 7.4. Before loading, APTES-modified MSMPs were dried under vacuum in a SpeedVac (Savant) for 8 h and then incubated in the liposome suspension for 30 min, at room temperature, under shaking at 1300 rpm, using 4 µL/1 × 10^7^ MSMPs. Fluorescence intensity of liposomes incubated with the MSMP was measured by spectrofluorimetry and the liposome amount was calculated by interpolation with the corresponding standard curves of fluorescence obtained for each liposomal formulation. Thereafter, liposome-containing MSMPs (i.e., MSVs) were centrifuged for 20 min at room temperature at 4000× *g*. Under this acceleration, MSMP and MSV quickly (few minutes) migrate in the tube bottom and form a pellet, while liposomes suspensions are unaffected by centrifugation below 15,000× *g*. The supernatant containing free liposomes was collected in a separate tube and the pellet containing the MSV particles was washed three times with the following procedure: MSVs were resuspended in 10-mM HEPES pH 7.4, using 0.5-mL/1 × 10^8^ MSV particles and subsequently centrifuged again at 4000× *g* for 10 min. Each time the supernatants containing a decreasing concentration of free liposomes were collected in a separate tube. After the washes, the MSVs were promptly used for further experiments, while the collected supernatants were used to measure the amount of free (non-encapsulated) liposomes. Fluorescence intensity (FI) of non-encapsulated liposomes in the supernatants, after each centrifugation (including the three washes), was measured by spectrofluorimetry with a PerkinElmer EnSpire microplate reader. Liposome amount was calculated by interpolation with standard curves of FI obtained for each liposomal formulation. Standard curves were obtained by linear regression of FI measures performed on serial dilutions (at known concentration) of liposome suspensions. The amount of liposomes loaded into the MSV was calculated by the difference between the total loaded quantity and the remaining free liposomes.

For the MSV assembly by lyophilization, the MSMPs incubated in the liposome suspension as described above (in this section) were frozen by plunging the tube containing the suspension in liquid nitrogen and then lyophilizing it under vacuum in an Edwards Modulyo lyophilizer for 10 h in the dark. After lyophilization, liposome-containing MSMPs (i.e., MSVs) were stored at 20 °C up to 1 month. Before use; lyophilized MSVs were rehydrated with H_2_O, by using 4 µL/1 × 10^7^ MSVs and centrifuged at 4000× *g* for 20 min at room temperature. The supernatant containing free liposomes was collected in a separate tube, the pellet containing the MSV particles was washed three times as described above. After each wash, the supernatant was collected in a separate tube. The collected supernatants were used to measure the amount of free (non-encapsulated) liposomes as described above. In addition. in this case, after washes, the MSVs were promptly used for further experiments. The amount of liposomes loaded into the MSVs was calculated as described above.

MSV assembly was evaluated by cytofluorimetry as reported elsewhere [11]. Briefly, 1 × 10^7^ MSV particles were resuspended in 500 µL of 10-mM HEPES pH 7.4 and analyzed by BD FACSCanto II and BD FACSDiva™ software (BD Biosciences). The events, properly gated on FSC-A/SSC-A and FSC-H/FSC-A plots, were analyzed for the mean fluorescence intensity (MFI) of the Oregon-Green-labeled liposomes contained in the MSV.

The MSV assembly was also evaluated by confocal microscopy as reported elsewhere [11]. Briefly, 1 × 10^7^ MSV particles were resuspended in 100 µL of 10-mM HEPES pH 7.4. One microliter of MSV suspension was spotted onto a microscopy glass slide, covered with a glass coverslip and observed with a Leica TCS SP5 confocal microscope by using a 63× oil-immersion objective and LAS AF software.

### 2.14. Release Kinetics of Liposomes from MSV

After washes, MSVs containing fluorescent liposomes were resuspended in PBS pH 7.4 using 0.5 mL/1 × 10^8^ MSV particles and were incubated at room temperature, under shaking at 1300 rpm. After 0.5, 1, 2, 4, 8, 24 h of incubation, MSVs were centrifuged at 4000× *g* for 10 min, and supernatants were collected in separate tubes. The amount of released fluorescent liposomes was calculated as described above (Section 2.11).

### 2.15. Size of Released Liposomes

After washes, MSVs were resuspended in PBS, using 0.5-mL/1 × 10^8^ MSV particles, and were incubated at room temperature, under shaking at 1300 rpm. After 1, 6, 12, 24, 48, 72 h of incubation, MSVs were centrifuged at 4000× *g* for 10 min and supernatants were collected in separate tubes. The supernatants were incubated at room temperature for 1 h under shaking at 2000 rpm with cycle of shaking by vortex for 1 min, every 15 min of incubation. The supernatants were then diluted 1:500 (*v*/*v*) in 10-mM HEPES pH 7.4 and Dh and PDI of released liposomes were measured as described above.

### 2.16. Confocal Microscopy of Cells Incubated with MSVs.

The wells of 8-well Falcon™ Chambered cell culture slides (Corning Life Sciences) were filled with 2% (*w/v*) porcine skin gelatin (Sigma-Aldrich) in PBS and left under laminar flow hood at room temperature for 10 min. The excess of gelatin was aspired, and the slides were left under a laminar flow hood at room temperature for 20 min to allow the gelatin to dry. HCT-116 cells were seeded in the wells of gelatin-coated chambered slides at a density of 2.5 × 10^4^ cells/cm^2^. After 24 h, cells were washed with PBS, and the culture medium was replaced with fresh medium containing 1 × 10^4^ MSVs/µL and 1 × 10^2^ MSVs/cells. After 24 h of incubation with MSVs, cells were washed twice with PBS and fixed in 4% (*w/v*) paraformaldehyde (Santa Cruz Biotechnology) in PBS, at room temperature for 10 min in gentle shaking. Cells were then washed twice with PBS and incubated with a solution of 0.1% (*v*/*v*) Triton X-100 (Sigma-Aldrich) in PBS for 5 min and then again washed twice in PBS. Cells were pre-incubated with PBS containing 1% (*w/v*) BSA (Sigma-Aldrich) for 30 min. A staining solution was prepared by diluting 1:40 (*v*/*v*) a stock solution (6.6 µM in methanol) of Alexa Fluor 546-conjugated phalloidin (Thermo Fisher Scientific) in PBS containing 1% (*w/v*) BSA (Sigma-Aldrich). The pre-incubation solution was then replaced with the staining solution in the chambered slide wells and left for 20 min, at room temperature, under gentle shaking, in the dark. Then the cells were washed twice with PBS and were incubated in PBS containing 1 µg/mL of 4’,6-diamidino-2-phenylindole dihydrochloride (DAPI) (Thermo Fisher Scientific) in PBS at room temperature for 5 min. After two washes with PBS, the chambers were removed from the slides and a coverslip glass was applied using ProLong™ Gold antifade mountant as mounting medium and antifade agent. The slides were observed by confocal microscopy using a Leica TCS SP5 confocal microscope (Leica Biosystems) with a 40× oil-immersion objective and the Leica “LAS AF” software. The experiments were performed in three independent replicates. For each analysis at least three microscope fields were acquired.

## 3. Results

### 3.1. Design of Particles

**Figure 1:**
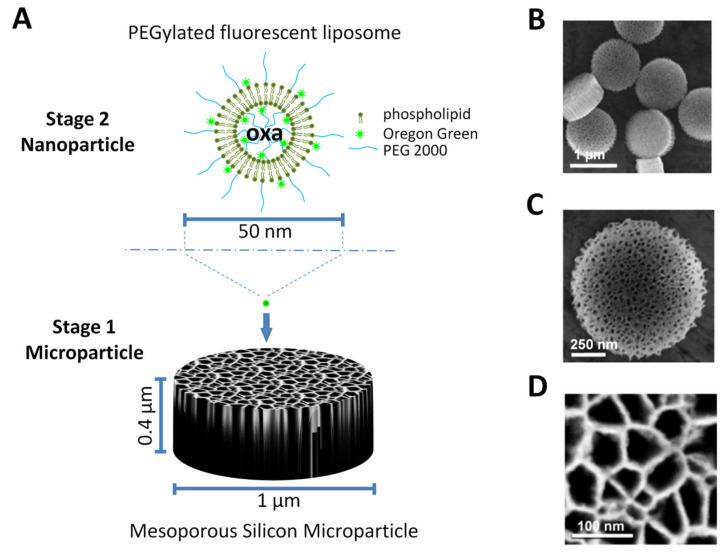
Mesoporous silicon microparticles (MSMP) and multistage vector (MSV). (**A**) Scheme of MSVs, showing the geometry and size of the MSMPs and liposomes; (**B**–**D**) scanning electron micrographs showing discoidal-shaped MSMPs 1 µm in diameter, 0.4 µm thick and with 60 nm pores; (**B**,**C**) two micrographs at different magnifications (reprinted (adapted) from *Journal of Controlled Release*, Vol. 158, Pages No. 148–155, *Rapid tumoritropic accumulation of systemically injected plateloid particles and their biodistribution*, van de Ven A.L. et al., Copyright (2012), with permission from Elsevier.) [12]; (**D**) detail of pores [reprinted (adapted) from *Accounts of Chemical Research, Vol. 44*, Pages No. 979–989, *Multistage Nanovectors: From Concept to Novel Imaging Contrast Agents and Therapeutics*, Godin B. et al., Copyright (2011), with permission from American Chemical Society [36].

We aimed to produce an MSV made up of an MSMP harboring oxa-loaded liposomes in its pores (Figure 1A). The MSMP is the first stage of the MSV, while oxa-loaded liposomes are the second stage. Our MSMP is disc-shaped, with 1-µm diameter (Figure 1B,C), 0.4-µm thickness (Figure 1C) and 60 nm (mean diameter) pores (Figure 1D). These MSMPs proved to be very effective in accumulating in the tumor environment, in previous studies [11,12]. We explored 16 liposomal formulations and two alternative procedures to identify liposomes compatible with the small MSMP pore size (Table 1). Each formulation was analyzed by DLS and LDE (Table 1). Indeed, five formulations (formulations 4, 10, 13, 15, 16) showed promising size (hydrodynamic diameter, Dh) and polydispersion index (PDI), able to fit the MSV pore size, besides having a sufficient surface charge (ζ-potential) to impede aggregation and to favor the interaction with the surface-charged silicon particle.

Oxa-loaded liposomes were then produced and analyzed by DLS and LDE (Table 1): only 15-oxa (DS-oxa) and 16-oxa (DO-oxa) had the required physicochemical parameters (Dh, PDI and ζ-potential), quite close to their empty counterparts (DO and DS). Thus, DO, DS, DO-oxa, and DS-oxa were the only formulations used for further experiments.

We also produced fluorescent labeled DO, DS, DO-oxa and DS-oxa formulations using 0.5% (*w/w*) DSPE conjugated with Oregon Green (OG) (Avanti Polar, AL, USA) in the lipid composition and we confirmed that Dh, PDI and ζ-potential were not modified using lipid fluorescent dye for the labeling (data not shown).

**Figure 2:**
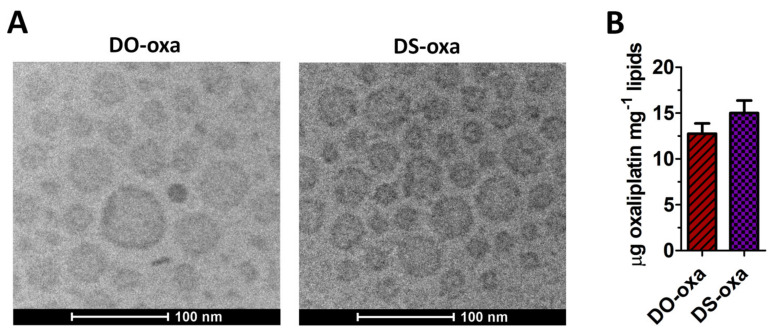
Oxaliplatin-loaded liposomes. (**A**) Cryo-transmission electron microscopy (cryo-TEM) micrographs of oxa-loaded liposomes. The cryo-TEM micrographs are representative of three independent experiments; (**B**) encapsulation efficiency of oxaliplatin inside liposomes. The amount of oxaliplatin loaded inside liposomes was measured by HPLC connected with a diode array detector. The entrapment efficiency was reported as μg of drug per mg^−1^ lipids. Oxa absorbance was measured at 249 nm. Error bars represent standard deviations. Statistical significance was calculated by one-way two-tailed *t*-test.

Cryo-TEM microscopy confirmed the size and PDI of the two selected oxa-loaded liposomes (Figure 2A). Reverse-phase HPLC analysis with DAD detection (Figure 2B) revealed a loading efficiency of 15.0 ± 1.4 µg oxa/mg lipids for DS-oxa and 12.7 ± 1.1 µg oxa/mg lipids for DO-oxa.

### 3.2. Effect of Liposomal Formulations in Cell Cultures

**Figure 3:**
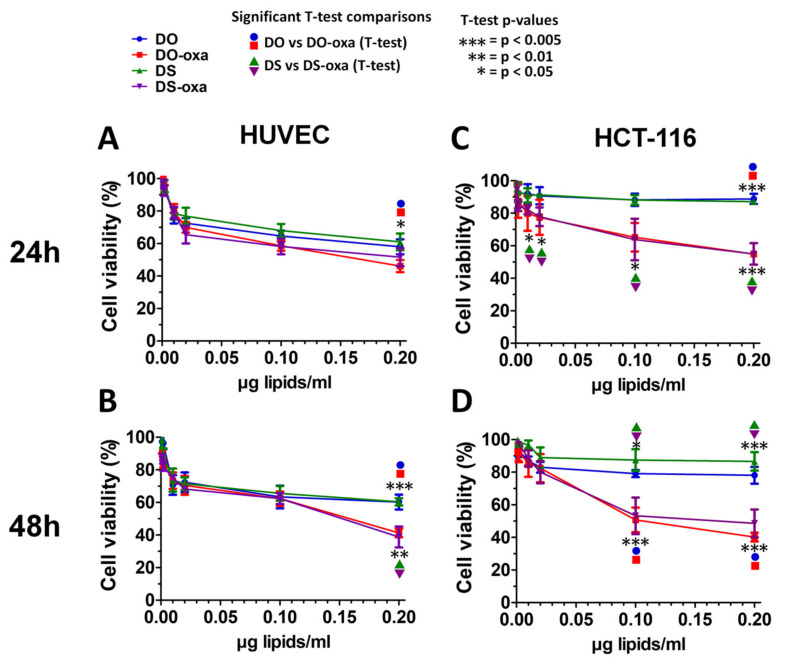
Viability of cells treated with liposomal formulations. Cell viability (%) was measured by 3-(4,5-dimethyl-2-thiazolyl)-2,5-diphenyl-2H-tetrazolium bromide (MTT) assay in human umbilical vein endothelial cells (HUVEC) at (**A**) 24 h and at (**B**) 48 h and in HCT-116 at (**C**) 24 h and at (**D**) 48 h. Controls are untreated cells and correspond to a cell viability of 100%. Experiments were performed in three independent replicates. Error bars represent standard deviations. Statistical significance was calculated by one-way two-tailed *t*-test.

To evaluate the effect of the selected liposomal formulations on cell viability, we performed time-course MTT assays (24, 48 h) on primary human umbilical vein endothelial HUVEC cells and human colorectal carcinoma cells HCT-116 treated with empty (DO, DS) and loaded liposomes (DO-oxa, DS-oxa) at different lipidic concentrations (0.001, 0.002, 0.01, 0.02, 0.1, 0.2 µg lipids/mL) in the culture medium (Figure 3). In HUVEC cells, both liposomal formulations showed a relevant degree of toxicity even at low concentration (0.02 µg/mL), without significant differences between loaded and unloaded NPs. After 24 h, cell viability ranged from 72% to 84% at 0.01 µg/mL, while at the highest concentration tested (0.20 µg/m), it dropped to 42–62%. At 48 h, this trend did not change significantly at the lowest concentrations, while the difference in cell viability between empty and loaded liposomes was significant (*p* < 0.005) for both DS-oxa and DO-oxa at the highest concentration tested. In HCT-116, oxa-independent toxicity of liposomes was less pronounced when compared with HUVEC cells, while oxa exerted a cytostatic/cytotoxic effect already after 24 h and at lower concentrations (Figure 3). The most relevant reduction of cell viability was reached, as expected, at the highest concentration (0.20 µg/mL) after 48 h and no relevant differences were detected between DO-oxa and DS-oxa, regardless of time and concentrations.

**Figure 4:**
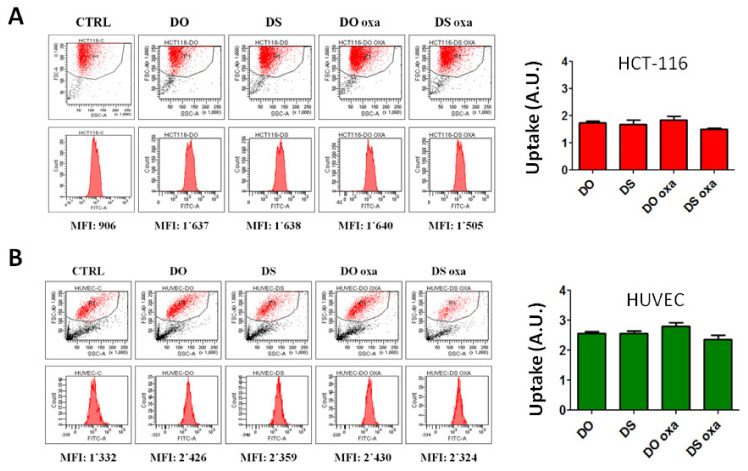
Endocytosis (uptake) levels of fluorescent liposomal formulations. Uptake was measured by cytofluorimetry, after three hours of incubation with 0.20 mg lipids mL culture, in (**A**) HCT-116 cells and (**B**) HUVEC cells. For the measurement, harvested cells were resuspended in PBS containing 1 mg/mL trypan blue, to quench extracellular fluorescence. Untreated cells were used as control (CTRL). Uptake is expressed in arbitrary units (A.U.) derived by mean fluorescence intensity (MFI) after background (CTRL mean fluorescence intensity (MFI), i.e., cell auto-fluorescence) subtraction and normalization with specific fluorescence intensity of each liposome type (see Materials and Methods). Experiments were performed in three independent replicates. Error bars represent standard deviations. Statistical significance was calculated by one-way two-tailed *t*-test.

To evaluate liposome internalization, HCT-116 and HUVEC cells were incubated with empty and loaded fluorescent liposomes for 3 h and then analyzed by cytofluorimetry to measure the fluorescence of internalized liposomes (Figure 4). The mean fluorescence intensity after background subtraction and proper normalization (see Materials and Methods) were reported as arbitrary units (A.U.) (Figure 2). Indeed, the uptake levels of the four formulations (DO, DS, DO-oxa, DS-oxa) did not differ from each other, in both the cell lines (Figure 4).

**Figure 5:**
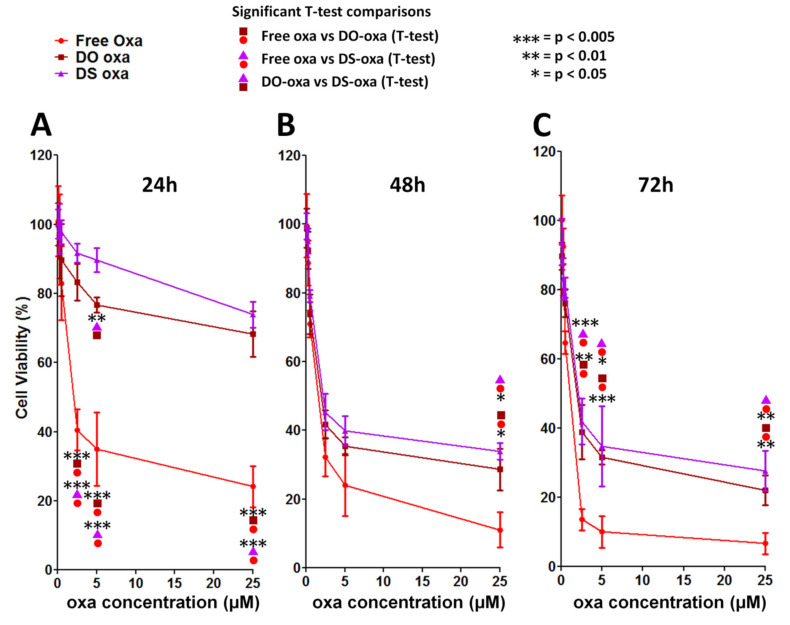
Viability of cells treated with free oxaliplatin and oxaliplatin-loaded liposomes. HCT-116 cell viability was measured by MTT assay at (**A**) 24 h, (**B**) 48 h and (**C**) 72 h. Experiments were performed in three independent replicates. Error bars represent standard deviations. Statistical significance was calculated by one-way two-tailed *t*-test.

To compare the cytotoxic/cytostatic effect of oxa-loaded liposomes with that of free oxa, we performed additional MTT assays in HCT-116 cells to calculate the corresponding IC_50_ (Figure 5 and Table 2). After 24 h of treatment, free oxa displayed an IC_50_ of 1.9 ± 0.7 µM (Table 2) while the viability of DO-oxa and DS-oxa was reduced by about 70% at the highest dose tested (25 µM) (Figure 5). Furthermore, after 48 h, the efficacy of free oxa (IC_50_ = 1.3 ± 0.6 µM) was higher than its encapsulated forms (Figure 5 and Table 2), but the difference was less relevant, mostly if compared with DO-oxa (IC_50_ = 1.6 ± 0.6 µM) (Table 2). However, after 72 h, no substantial improvements were noted for DO-oxa and DS-oxa, while the gap with the more effective free oxa (IC_50_ = 0.8 ± 0.3 µM) became again significant (Figure 5 and Table 2).

### 3.3. Multistage Vector Assembly

**Figure 6:**
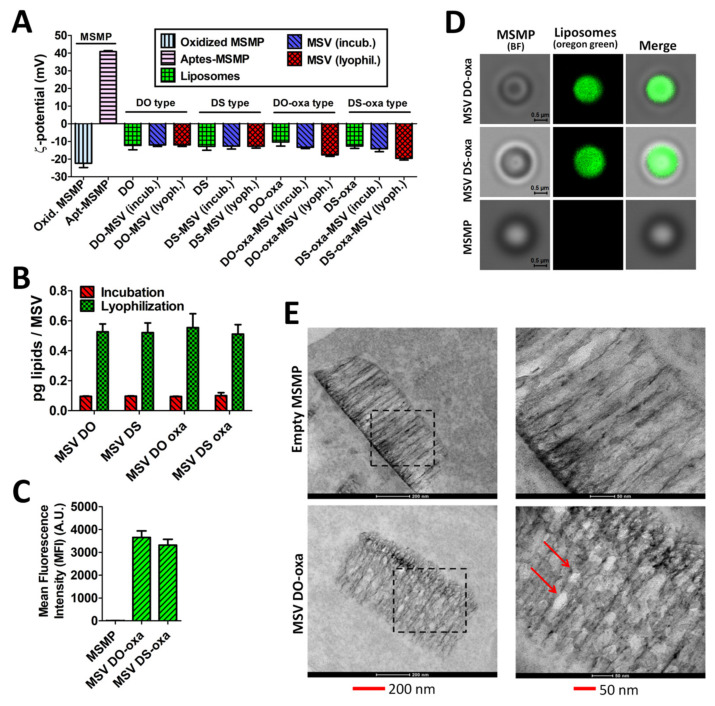
MSV assembly. (**A**) ζ-potential analysis of the empty MSMPs, selected liposomes and assembled MSVs; (**B**) amount of fluorescent liposomes loaded into the MSMP and measured by spectrofluorometer; (**C**) relative levels of mean fluorescence intensity (MFI) of liposomes loaded into the MSMP (i.e., MSV assembly) measured by cytofluorimetry (see also Figure S2); (**D**) qualitative analysis of MSV assembly by confocal microscopy; (**E**) TEM micrographs of the MSV. An empty MSMP and MSV DO-oxa are showed. Experiments were performed in three independent replicates. Error bars represent standard deviations. Statistical significance was calculated by one-way two-tailed *t*-test. Abbreviations: incub.—incubation; lyoph.—lyophilization; incub. and lyoph. refer to two methods for MSV assembly (see Materials and Methods section); A.U.—arbitrary units.

To promote the interaction of the selected liposomes with the S1MPs, the silicon surface was modified by conjugation with APTES which confers a positive surface charge [18] (Figure 6A) to the microparticles. The subsequent loading of negatively charged liposomes into the S1MP pores caused the expected reversal in the surface charge of the MSV (Figure 6A). Indeed, the surface charge modification was particularly relevant since APTES-modified S1MP had a ζ-potential of +40.8 ± 0.5 mV, while assembled MSVs had a ζ-potential in a range from −11.9 ± 0.9 to −19.6 ± 1.0 mV.

To load the liposomes into the MSMPs, we applied two different methods: (i) incubation of dry MSMPs in concentrated (2 mg/mL) liposomal suspension; (ii) incubation followed by lyophilization. Interestingly, MSV DO-oxa and MSV DS-oxa, obtained with the lyophilization method, were more negatively charged (−17.6 ± 0.9 and −19.6 ± 1.0 mV, respectively) than either the particles assembled in suspension or the corresponding empty liposomes DO-oxa and DS-oxa (−10.3 ± 2.3 and −12.6 ± 1.4 mV, respectively).

We also measured the amount of loaded liposomes by spectrofluorimetry (Figure 6B). Using the lyophilization method, we were able to load more than 5-fold liposomes (0.511 ± 0.064–0.554 ± 0.092 pg lipids/MSV particle) than using the method based on simple incubation (0.943 ± 0.002–0.100 ± 0.020 pg lipids/MSV particle) (Figure 6B). However, loading efficiency did not differ significantly among the four MSVs when they were obtained using the same assembly method (Figure 6B).

We chose the lyophilization method as the elective procedure. Hence, all the subsequent experiments were performed by assembling the MSV via lyophilization, unless otherwise specified.

Cytofluorimetry (Figure 6C; Figure S2), confocal microscopy (Figure 6D) and TEM analysis (Figure 6E) demonstrated the successful internalization of loaded liposomes into the MSV by lyophilization.

### 3.4. Release of Liposomes from the Multistage Vector

**Figure 7:**
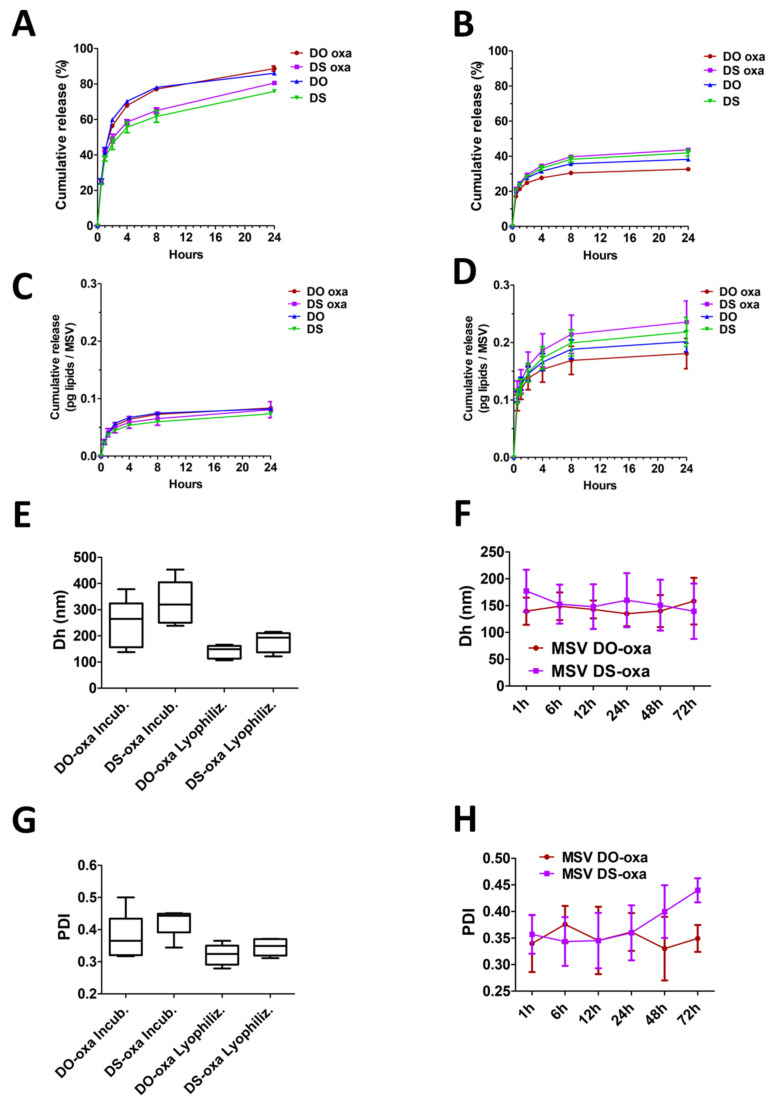
Release of liposomes from the MSV. Kinetics of cumulative release of liposomes from the MSV; percentage of liposomes released after loading (**A**) by incubation and (**B**) by lyophilization; picograms of lipids released after loading (**C**) by incubation and (**D**) by lyophilization. Experiments were performed in three independent replicates; (**E**) hydrodynamic diameter (Dh, nm) of oxa-liposomes released from the MSV after 1 h; (**F**) Dh of lyophilized liposomes released from the MSV in a time-course analysis; (**G**) polydispersion index (PDI) of oxa-liposomes released from the MSV after 1 h; (H) PDI of lyophilized liposomes released from the MSV in a time-course analysis. Experiments were performed in five independent replicates. Error bars represent standard deviations.

After the MSV assembly, we evaluated the cumulative release of fluorescent liposomes from the MSV (Figure 7). In general, the four formulations (DO, DS, DO-oxa and DS-oxa) did not show different release kinetics when generated with the same MSV assembly method. In all cases the curves had a two-phase trend, namely, an initial quick release followed by a phase in which the liposomes were released much more slowly. However, there were substantial quantitative differences between assembly by incubation and assembly by lyophilization. In fact, the release of liposomes, expressed as released percentage of the total loaded amount, was faster from the MSVs assembled by incubation compared to lyophilized MSVs (75.80% ± 0.39% to 88.68% ± 1.45% vs. 32.60% ± 0.74% to 43.64% ± 1.12% after 24 h) (Figure 7A,B). However, after 24 h, the total amount of liposomes released by lyophilized MSVs (0.181 ± 0.026 to 0.236 ± 0.037 pg lipids/MSV particle) was significantly higher than the MSVs prepared with the incubation method (0.073 ± 0.001–0.084 ± 0.002 pg lipids/MSV particle) (Figure 7C,D), due to the dramatic difference of loading efficiency between the two protocols (Figure 6B). Moreover, among the MSVs assembled by lyophilization, MSV DO-oxa had a lower rate of release than MSV DS-oxa (0.181 ± 0.026 vs. 0.236 ± 0.037 pg lipids/MSV particle after 24 h).

We then measured the size of the liposomes released after 1 h by DLS (Figure 7E,F). In both the applied loading methods, we registered an increase in the diameter of released liposomes, with higher values for those obtained by means of the incubation assembly protocol: in fact, MSV DO-oxa and MSV DS-oxa, assembled by incubation, after 1 h, released liposomes with a mean size of 245 ± 94 and 326 ± 85 nm, respectively, while their counterparts obtained by lyophilization, exhibited a mean size of 140 ± 25 and 177 ± 39 nm, respectively. All the released liposomes showed a relative dimensional homogeneity with a mean PDI below 0.45 (Figure 7G,H).

We also performed a time-course analysis of the size of liposome released by lyophilized MSV. This analysis showed no significant differences among the various time points and between the two formulations tested (DO-oxa and DS-oxa) but confirmed a substantial increase in the size of released liposomes if compared with their original size before the loading into the S1MP. The released liposomes showed a relative dimensional homogeneity at every time point with a mean PDI below 0.45 (Supplemental Figure S1B).

### 3.5. Effects of the Multistage Vector in Cell Culture

**Figure 8:**
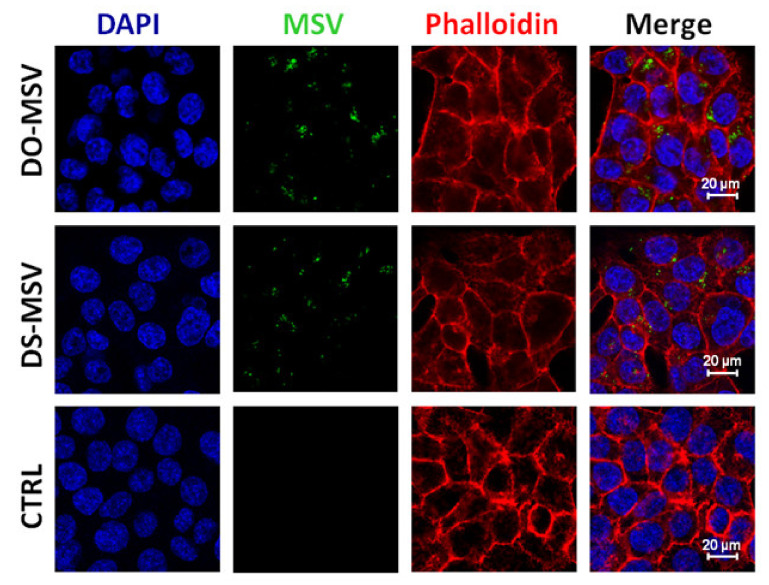
Confocal microscopy images of cells treated with MSV-oxaliplatin-loaded liposomes. HCT-116 cells were incubated for 24 h with 1 × 10^4^ particles/µL medium, 100 particles/cell. Untreated cells served as control (CTRL).

We treated HCT-116 with fluorescent MSV-DO-oxa and fluorescent MSV DS-oxa (assembled by lyophilization), and after 24 h, we measured the MSV fluorescence inside the cells by confocal microscopy (Figure 8). After 24 h of incubation, most of the fluorescent liposomes remained inside the MSV, as shown by release dynamics (Figure 7B). Thus, in this experiment we assigned the green fluorescent signal (Figure 8) to liposomes still inside the MSV and not to released liposomes. This analysis indicates that HCT-116 cells easily internalize the entire MSV particle (together with its liposomal payload), as shown in other cellular models (Figure 6E) [11,18].

**Figure 9:**
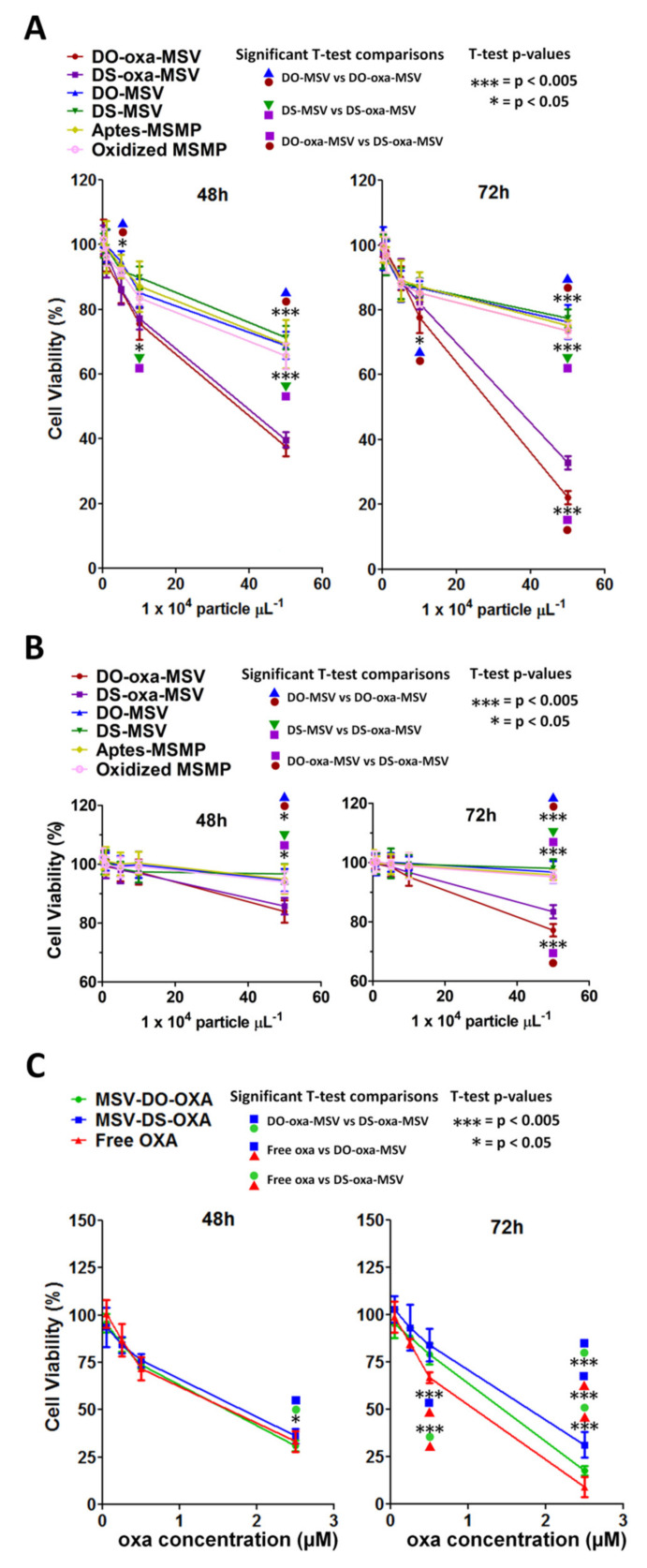
Cell viability effects of MSVs. MTT assay at 48 and 72 h in (**A**) HCT-116 and (**B**) HUVEC incubated with different MSVs and empty S1MPs with respect to particle concentration; (**C**) MTT assay at 48 and 72 h in HCT-116 incubated with MSVs and free oxa with respect to oxa concentration. Experiments were performed in three independent replicates. Error bars represent standard deviations. Statistical significance was calculated by one-way two-tailed *t*-test.

To measure the cytotoxicity of the MSVs and of the empty MSMPs, we evaluated the viability of cells treated with MSV DO-oxa, MSV DS-oxa, MSV DO, MSV DS and the empty MSMP (in both oxidized and APTES-modified form) (Figure 9A,B). In HCT-116 cells, the cytotoxicity of the empty MSMPs, MSV DO and MSV DS was already evident (66% ± 4% to 71% ± 4% cell viability) at 48 h, with the highest tested concentration (50 × 10^4^ particles/µL medium), whereas, as expected, MSV DO-oxa and MSV DS-oxa caused a significantly higher reduction (38% ± 3% to 40% ± 2% cell viability) (Figure 9A). After 72 h, the cytotoxic effect of MSV DO-oxa and MSV DS-oxa was even higher, with a significant difference between MSV DO-oxa (22 ± 2% cell viability) and MSV DS-oxa (33 ± 2% cell viability) at a concentration of 50 × 10^4^ particles/µL medium (Figure 9A).

In HUVEC cells, empty S1MPs, MSV DO and MSV DS did not show evident cytotoxicity at 48 h, while MSV DO-oxa and MSV DS-oxa caused a slight, but significant reduction of cell viability (84% ± 4% to 86% ± 3%) only at the highest tested concentration (50 × 10^4^ particles/µL medium) (Figure 9B). After 72 h, the effect of empty S1MPs, MSV DO, and MSV DS remained stable, whereas at the highest tested concentration (50 × 10^4^ particles/µL medium) of MSV DO-oxa and MSV DS-oxa, showed a more pronounced effect than that observed at 48 h, but less cytotoxic if compared with that obtained in HCT-116 cells. Moreover, at 72 h there was a significant difference between MSV DO-oxa (77 ± 2% cell viability) and MSV DS-oxa (83 ± 2% cell viability) at the concentration of 50 × 10^4^ particles/µL medium (Figure 9B).

Finally, we compared the effect of oxa encapsulated in MSV with that of free oxa (Figure 9C) in HCT-116 cells by calculating the IC_50_ parameter (Table 3). We observed that after 48 h, the cytostatic/cytotoxic effect of loaded MSVs was comparable to that of free oxa, causing a drop in cell viability to 31% ± 3% to 36% ± 4% at a 2.5-µM oxa concentration. At this time point, no statistically significant difference in the IC_50_ was observed among free oxa, MSV DO-oxa and MSV DS-oxa (IC_50_ range: 1.1–1.8 µM). After 72 h of incubation, free oxa was significantly more effective than its encapsulated forms (9 ± 5% cell viability) (Table 3) at 2.5 µM oxa. At the same concentration also the difference in cell viability between MSV DO-oxa and MSV DS-oxa was significant (18 ± 2% vs. 31 ± 7%, respectively). At this time point, the corresponding IC_50_ showed statistically significant differences among the three systems: free oxa (0.7 ± 0.1 µM), MSV DO-oxa (1.0 ± 0.1 µM) and MSV DS-oxa (1.5 ± 0.3 µM).

## 4. Discussion

The MSMPs used in this study are the most stable first stage microparticles with the largest mean pore size able to host small unilamellar liposomes. Liposomes were designed and synthesized using different combinations of rigid, fluid and PEGylated lipids to obtain formulations with different geometric properties and curvature radii of the bilayer. These properties of the phospholipids depend on their molecular geometry that is influenced by the cross-sectional area of the head group, by the length of acryl chains and by the packing parameter [49]. The curvature radius of the phospholipid bilayer affects the membrane structure and its rigidity; as a consequence, the lamellar bilayer produces lateral pressure that changes the thermodynamic forces of lipids and generates overall superficial tension of liposomes [50].

The production of stable liposomes smaller than 60 nm is a challenge because the extreme surface curvature can counteract the intermolecular forces which stabilize the supramolecular structure of the lipid bilayer [49]. Liposomes smaller than 60 nm are sometimes similar to micelles and/or bicelles where a single or a bicontinuous lipid layer surrounds a small aqueous cavity thereby creating a unique supramolecular structure with a hydrophobic bilayer [51,52]. This effect depends on the shape of phospholipids as well as on the intra- and intermolecular forces between phospholipids and water surrounding hydrophilic structure (head group) of macromolecules [50]. The resulting energy can force hydrophobic and hydrophilic layers to combine into a single layer without discriminating between aqueous and lipophilic compartments. Such a phenomenon occurs when, in this configuration, the differences of free energy of phospholipids are not significant and depend on the saturated and unsaturated nature of phospholipids, self-assembled in the lipid bilayer [50].

The preparation procedure can also affect the balance between the surface curvature radius and the intermolecular forces of phospholipids without altering the resulting energy of the supramolecular structure of liposomes [49]. Consequently, we explored several lipidic compositions and different procedures; we found that the experimental method that resulted in the most suitable liposomes (in terms of size and PDI) was extrusion through 30 nm-pore polycarbonate membranes. In particular, the higher transition temperature of the selected liposomal formulations, due to the specific lipid composition, hinted a more rigid supramolecular structure able to drive a more precise size in the extrusion process [49]. Thanks to this feature, liposomes are suitable for MSV pores, without altering their physicochemical properties and maintaining their native spherical structure. In particular, we used rigid lipids, like DSPC, that do not need to be stabilized with cholesterol and make stable liposomes alone [53] and fluid lipids, like DOPC, that have a transition temperature below −4 °C (Avanti Polar Lipids, Inc.; Alabaster, AL, USA) and need cholesterol to stabilize the lipid bilayer [54]. PEGylated lipids were used to stabilize further the liposomal formulations particularly in the case of DO liposomes. In fact, PEG groups form a steric barrier around the liposomal surface that limits MPS uptake, the aggregation and the modification of liposome surface properties as well as enzymatic degradation in biologic fluids. Thus, these effects preserve the physicochemical and biopharmaceutical properties of liposomes when they are used in vitro (e.g., incubated in plasma or suspended in cell culture media) or in vivo (e.g., intravenously injected) [55].

The main drawback ascribed to such extra-small liposomes is a lower efficiency in oxa encapsulation (about 10 µg of drug per mg lipids) if compared with nanovesicles above 100 nm (about 100 µg of drug per mg lipids), confirming that the entrapment efficiency of payloads depends on liposome sizes [56]. Furthermore, the presence of fatty acids in the lipid structure makes the bilayer more fluid and modifies the relative surface forces between phospholipids, thereby generating interface tensions and increasing the curvature stress of liposomes. This effect modifies the supramolecular structure of lipid membranes [57], thus increasing the drug leakage during the extrusion process, thereby leading to a decreased drug encapsulation efficiency. Nevertheless, despite the relatively low encapsulation efficiency, our liposomal formulations exerted a reasonable oxa-dependent cytostatic effect in colon cancer cells [58,59].

On the other hand, the oxa-induced reduction in cell viability appeared less relevant in primary endothelial cells, in agreement with other studies [60].

Our liposomes were able to enter the MSMP pores thus leading to a correct MSV assembly. In particular, the liposomes were loaded inside MSMPs as fresh colloidal suspension or lyophilized products. The lyophilization strategy greatly improved the loading of the liposomes inside the 60-nm pores of MSMPs; in fact, this procedure led to the loading of a 5-fold larger amount of liposomes than that obtained by a simple incubation. Such a difference can be partly due to the larger hydrodynamic radius of liposomes in suspension versus lyophilized liposomes [61]. During the lyophilization process, the latter undergo transition to a solid state, like drug powder, and, as a consequence, they better fit the 60-nm pores of MSMPs. Moreover, the lyophilization strategy exploits the driving force of liposomal embedding. Indeed, the gradient of concentration between the external compartment and the internal cavities of MSMP pores favors passive loading of lyophilized liposomes. Conversely, when in suspension, liposomes have a higher hydrodynamic radius due to water molecules that form the surface hydration layer. The larger size of liposomes [62] hampers their complete loading within the MSMP.

The kinetics of liposome release from the MSV had a two-phase trend, namely, an initial quick release followed by a phase in which the liposomes were released much more slowly. The release of NPs from a variety of MSV platforms was widely investigated in last decade [11,13,46]. The release from the MSV is essentially due to two main phenomena: i) the concentration gradient of NPs loaded within the MSMP pores; ii) the degradation of the silicon structure of the MSMP. In fact, we previously reported that the MSMP is quickly degraded in aqueous solutions in which it is completely dissolved after few days [10,11,18,46,47]. However, we also reported that the embedding of the MSMP with soft materials like lipids, proteins and polymers, can significantly delay its degradation [11,18]. We may hypothesize that this phenomenon occurs also in the case of the herein studied MSV platform and may explain the relatively slow liposome release observed in this study.

The two different protocols for liposome embedding into the MSMPs used in this study not only determined a different outcome in terms of the amount of loaded liposomes, but also affected the subsequent release from the MSMPs: the percentage of released liposomes was significantly higher, after 24 h, in the case of liposomes loaded in suspension; nevertheless, the absolute amount of released liposomes was very higher in the case of liposomes loaded in suspension.

We can suppose that lyophilized liposomes, when rehydrated inside the MSV, become larger than its pores, which have an average diameter of 60 nm. This phenomenon generates a depot effect inside the MSV that induces a slower release of the loaded liposomes over a shorter time and consequently increases the release of the payload after 24 h compared to liposomes loaded in MSV as suspension. The depot effect and the bulk release of liposomes observed after 24 h may depend on the lag-time necessary to move through the narrower MSV pores following a gradient of concentration and hampers the coming out of the liposomes. In fact, lyophilized liposomes, after internal hydration, are forced to squeeze through the pores of MSV thus slightly modifying their structure. In fact, The MSV assembly caused a significant increase in the size of released liposomes. In particular these latter were larger in the case of the assembly by suspension strategy than in the case of the assembly by lyophilization strategy. This finding is a further advantage of the assembly by lyophilization.

Indeed, the changes in the size of released liposomes are likely due to the rearrangements of the lipidic vesicles within the narrow MSMP pores. When liposomes, as a suspension, are confined in the pores of MSMP particles, they shift their lateral pressure toward the membrane interspace and generate an increase of the tension in the central portion of phospholipid acyl chains that form the lipid bilayer. This effect causes a significant increase in the curvature stress of the bilayer and between the two monolayer sheets forming the structure of liposomes [49]. This increase modifies the surface tension of phospholipids that changes their shape, thus forming a less deformable and thermodynamically stable bilayer as well as some aggregates [49]. The presence of saturated and unsaturated phospholipids in the lipid bilayer, as well as the stabilizing effect of PEG moieties, can positively or negatively enforce this effect [49]. We may speculate that, under these conditions, liposomal suspensions increased the pressure per phospholipid unit necessary to the liposomes to come out from the pores of MSV particles. This effect mimics the forces applied on liposomes when they are extruded through polycarbonate membranes during the extrusion procedure. When this pressure exceeds the tension limit of phospholipids, it can allow fragmentation and a rearrangement of liposomes, thus producing medium-sized unilamellar liposomes similarly to what occurs when extrusion techniques are combined with the freeze–thaw method [63].

However, the DO liposomes showed more limited dimensional changes than the DS liposomes. This difference depends on the physicochemical properties of lipids making liposomes. DO-oxa has oleic acid-derived lipids in its structure compared to DS-oxa, which has stearic acid- derived lipids in the bilayer structure. Oleic acid lipids are more fusogenic and increases the fluidity of lipid bilayer compared to rigid stearic acid lipids that make stiff bilayers and in a gel-like configuration [64]. The different Tm of phospholipids influences the overall rigidity of the liposomal bilayer and possibly the amount of intact released liposomes, as well as aggregated lipid fragments [65]. Furthermore, the Tm of the phospholipids can modulate the transition of resulting liposomes from gel-like to liquid crystal state thus affecting their structure and shape during the release through the MSV pores.

When the assembled MSV was incubated in cell culture, it was easily internalized by cells [11,17,18], and DO-oxa and DS-oxa exerted a significant oxaliplatin-dependent effect in terms of reduction of viability of colon cancer cells. In fact, after 48 h of treatment, both types of oxa-liposome-loaded MSVs had an IC_50_ equivalent to that of free oxaliplatin. Nevertheless, oxaliplatin-independent intrinsic cytotoxicity of the empty S1MPs and of the MSVs containing empty liposomes is quite relevant in HCT-116 cells. Therefore, the effect of oxa-liposome-loaded MSVs may be the sum of the impacts of both the drug and the vector. However, this effect became particularly evident at the highest MSV concentration tested, which was 5-fold or higher than that used in other studies [11,66].

Furthermore, overall evidence is that drug-free MSVs and S1MPs particularly affect HCT-116 viability while they can be relatively well tolerated by other cell types. In fact, empty S1MP and MSV containing empty liposomes did not have a similar effect in HUVEC cells [66]. On the other hand, MSV embedded with oxa-liposomes also reduced HUVEC cell viability albeit to a much lesser extent than HCT-116, which confirms the relatively poor cytostatic effect of oxa in HUVEC cells [59].

The cytostatic efficacy of oxa-liposome-embedding MSV in CRC cell culture did not exceed that of free oxa. However, it is widely agreed that drug encapsulation hardly enhances the therapeutic index, even in vivo, while the expected overall effects can consist mainly in a more selective biodistribution, in more favorable pharmacokinetics and in fewer adverse side effects [67]. The overall characteristics of our oxa-liposome-loaded MSVs—and in particular of the formulation termed “MSV DO–oxa”, can be considered a promising tool for therapeutic strategies. This study of the characteristics and limits of extremely small liposome within the context of nanomedicine strategies, may suggest hints about the future use of such NPs in drug delivery applications. Moreover, the improvements we report related to the loading of liposomes into the pores of MSMPs could be useful for the production of enhanced versions of MSVs, whereas MSMPs with slightly larger pores (e.g., 80–100 nm) could allow the use of liposomes with less extreme features and a higher drug-loading efficiency.

## 5. Conclusions

In this work, we developed a MSV for the delivery of oxaliplatin and thus potentially useful for CRC treatments; this vector was based on an MSMP with previously proven preclinical efficacy in terms of tumor targeting capability in different cancer contexts. Various formulations of extra-small liposomes where tested in order to select the ones which could efficiently be used in MSV assembly; two oxaliplatin liposomal formulations were then selected and their loading into the MSV was optimized.

The main limit of this procedure shows a limitation due to a relative low efficiency of drug encapsulation into the extra-small liposomes, and to the dimensional changes the liposomes underwent after the release from the MSV. Both these negative effects were partially compensated in the MSV assembly, by the use of a novel lyophilization strategy; in fact, this procedure permitted to reach a higher concentration of liposomes per MSV particle. Furthermore, also the size change of liposomes, after their transit within MSV pores, was smaller in the case of lyophilized liposomes allowing a better release and yield of therapeutic bullets into the desired cells.

The important advantage of this study was that oxa-MSV showed reasonable impairment of CRC cell viability when compared with unencapsulated oxa; this can encourage the use of this MSV in future tests in vivo for CRC therapy, given the previously reported ability in tumor targeting of the MSMP hereby used for the MSV assembly. On the other hand, the studies on the production of extra-small liposomes and on the optimization of their loading into the pores of the MSMP represent a valuable enrichment of the knowledge about these matters giving useful hints on the design of future drug encapsulation and delivery strategies.

## Figures and Tables

**Table 1:** Liposomal formulations. Hydrodynamic diameter (Dh) and polydispersity index (PDI) were measured by dynamic light scattering (DLS). ζ–potential was measured by laser Doppler electrophoresis (LDE). Experiments were performed in three independent replicates. Results are the average of five measurements per replicate (mean ± standard deviation).

Form.	Composition	Short Name	Procedure	Tm	Dh	PDI	ZP
1	DOPC/DSPEmPEG_2000_ (7/3 molar ratio)	–	Extrusion 50 nm/Sonication	37 °C	58.96 ± 1.21	0.369 ± 0.02	−0.57 ± 0.03
2	PC/DSPEmPEG_2000_ (7/6 molar ratio)	–	Extrusion 50 nm/Sonication	45 °C	55.20 ± 2.25	0.363 ± 0.09	−1.59 ± 0.20
3	DOPC/DSPEmPEG_2000_ (7/5 molar ratio)	–	Extrusion 50 nm/Sonication	45 °C	55.5 ± 2.1	0.340 ± 0.03	−2.15 ± 0.30
4	DOPC/DSPEmPEG_2000_ (7/6 molar ratio)	–	Extrusion 50 nm/Sonication	37 °C	45.33 ± 1.2	0.476 ± 0.12	0.858 ± 0.10
5	PC/DSPEmPEG_2000_ (7/6 molar ratio)	–	Extrusion 50 nm/Sonication	45 °C	52.95 ± 2.1	0.467 ± 0.10	−1.59 ± 0.20
6	PC/DSPEmPEG_2000_ (7/5 molar ratio)	–	Extrusion 50 nm/Sonication	45 °C	53.46 ± 2.10	0.377 ± 0.09	−4.83 ± 1.10
7	DOPC/DSPEmPEG_2000_ (7/6 molar ratio)	–	Extrusion 50 nm/Sonication	37 °C	45.22 ± 1.90	0.471 ± 0.12	−6.87 ± 1.30
8	PC/DSPEmPEG_2000_ (7/6 molar ratio)	–	Extrusion 50 nm/Sonication	45 °C	53.03 ± 0.90	0.491 ± 0.13	−8.72 ± 0.90
9	PC/DSPEmPEG_2000_ (7/5 molar ratio)	–	Extrusion 30 nm	45 °C	52.93 ± 1.25	0.271 ± 0.12	−9.47 ± 1.70
10	PC/DSPEmPEG_2000_ (7/6 molar ratio)	–	Extrusion 30 nm	45 °C	47.76 ± 3.90	0.273 ± 0.09	−15.0 ± 2.10
11	DPPC/Chol/PE/DSPEmPEG_2000_ (7/7/4/1 molar ratio)	–	Extrusion 50 nm/Sonication	50 °C	55.58 ± 1.90	0.194 ± 0.09	−12.63 ± 1.23
12	PC/Chol/PE/DSPEmPEG_2000_ (7/7/4/1 molar ratio)	–	Extrusion 50 nm/Sonication	50 °C	54.16 ± 2.10	0.197 ± 0.08	−11.06 ± 0.30
13	DOPC/DPPC/DSPEmPEG_2000_ (7/4/3 molar ratio)	–	Extrusion 50 nm/30 nm	40 °C	51.5 ± 8.90	0.172 ± 0.10	−5.40 ± 5.0
14	DPPC/DPPS/Chol (7/4/7 molar ratio)	–	Extrusion 50 nm	50 °C	64.93 ± 1.21	0.053 ± 0.02	−41.94 ± 2.90
**15**	DSPC/DSPEmPEG_2000_ (7/3 molar ratio)	**DS**	Extrusion 30 nm	60 °C	26.3 ± 1.2	0.578 ± 0.02	−10.51 ± 0.18
**16**	DSPC/DOPC/DSPEmPEG_2000_ (7/1/2 molar ratio)	**DO**	Extrusion 30 nm	60 °C	54.46 ± 3.03	0.209 ± 0.03	−9.84 ± 0.40
4 oxa	DOPC/DSPEmPEG_2000_ (7/6 molar ratio) + oxa	–	Extrusion 30 nm	42 °C	64.5 ± 1.1	0.239 ± 0.91	−6.17 ± 1.23
10 oxa	PC/DSPEmPEG_2000_ (7/6 molar ratio) + oxa	–	Extrusion 30 nm	50 °C	75.2 ± 1.2	0.365 ± 0.14	−4.25 ± 1.60
13 oxa	DOPC/DPPC/DSPEmPEG_2000_ (7/4/3 molar ratio) + oxa	–	Extrusion 30 nm	50 °C	64.65 ± 5.15	0.098 ± 0.01	−5.35 ± 2.40
**15 oxa**	DSPC/DSPEmPEG_2000_ (7/3 molar ratio) + oxa	**DS-oxa**	Extrusion 30 nm	60 °C	44.25 ± 4.25	0.315 ± 0.04	−10.95 ± 0.4
**16 oxa**	DSPC/DOPC/DSPEmPEG_2000_ (7/1/2 molar ratio) + oxa	**DO-oxa**	Extrusion 30 nm	60 °C	42.60 ± 3.80	0.251 ± 0.01	−9.41 ± 1.95

Abbreviations: Form—formulation; PC—chicken egg L-α-phosphatidylcholine; Chol—cholesterol; PE—1,2-dipalmitoyl-sn-glycero-3-phosphatidylethanolamine; DSPEmPEG_2000_—1,2-distearoyl-sn-glycero-3-phosphoethanolamine-N-[methoxy(polyethylene glycol)-2000]; DOPC—1,2-dioleoyl-sn-glycero-3-phosphocholine; DPPC—1,2-dipalmitoyl-sn-glycero-3-phosphocholine; DSPC—1,2-distearoyl-sn-glycero-3-phosphocholine; oxa—oxaliplatin; Dh—hydrodynamic diameter (nm); ZP—ζ–potential (mV); MSV—multistage vector; oxa—oxaliplatin. DS—selected liposomal formulation containing DSPC as main lipid. DO—selected liposomal formulation containing DSPC and DOPC as main lipids.

**Table 2:** Inhibitory concentration (IC_50_) of free oxaliplatin and oxaliplatin-loaded liposomes. IC_50_ values were calculated by GraphPad Prism software through interpolation with curves of Figure 5. Statistical significance was calculated by the *t-*test; comparisons DO-oxa vs. DS-oxa, DO-oxa vs. Free Oxa, DS-oxa vs. free oxa were significant at 48 h and 72 h (*p* < 0.05).

IC_50_			
**Time (h)**	**Free oxa**	**DO-oxa**	**DS-oxa**
24	1.9 ± 0.7 μM	N.A.	N.A.
48	1.3 ± 0.6 μM	1.6 ± 0.6 μM	2.2 ± 0.8 μM
72	0.8 ± 0.3 μM	1.6 ± 0.7 μM	1.9 ± 0.8 μM

Abbreviations: N.A.—not available.

**Table 3:** Inhibitory Concentrations (IC_50_) of oxa-liposome-MSV and free oxa. IC_50_ values were calculated by GraphPad Prism software through interpolation with the curves of Figure 9. Statistical significance was calculated by *t-*test; comparisons DO-oxa vs. Free Oxa, DS-oxa vs. free oxa were significant only at 72 h (*p* < 0.05).

Time (h)	Treatment	IC_50_ (μM)	SD (μM)
48	Free oxa	1.3	± 0.2
	MSV DO-oxa	1.2	±0.1
	MSV DS-oxa	1.5	±0.3
72	Free oxa	0.7	±0.1
	MSV DO-oxa	1.0	±0.1
	MSV DS-oxa	1.5	±0.3

Abbreviations: SD—standard deviation.

## Supplementary Materials

The following are available online at https://www.mdpi.com/1999-4923/12/6/559/s1, Table S1: HPLC gradient elution for the analysis of oxa in liposomal formulations, Table S2: Recovery values obtained with the weighted-linear least-squares regression analysis of six independent eight non-zero concentration points of oxa with liposomal formulations, Figure S1: HPLC chromatograms of oxa (A), oxa and IS (B), and oxa-loaded liposomes (C) after separation and quantification by HPLC analysis (Section 2.8 of Materials and Methods), Figure S2: Cytofluorimetryic analysis of multistage vector (MSV) assembly. MSV particles were resuspended in 10 mM Hepes pH 7.4 and analyzed by BD FACSCanto II and BD FACSDiva™ software (BD Biosciences, Franklin Lakes, NJ, USA). The events, properly gated on FSC-A/SSC-A and FSC-H/FSC-A plots, were analyzed for the mean fluorescence intensity (MFI) of the oregon-green-labeled liposomes contained in the MSV. Empty mesoporous silicon microparticles (MSMP) were used as a negative control. The figure is representative of one out of three independent experimental replicates.
