# Supplementary material for: Liposome-Embedding Silicon Microparticle for Oxaliplatin Delivery in Tumor Chemotherapy
Source: Pharmaceutics. 2020 Jun 17;12(6):559. doi: 10.3390/pharmaceutics12060559 (PMC7355455; doi:10.3390/pharmaceutics12060559)
Supplement: Supplementary file 1 [file pharmaceutics-12-00559-s001.pdf]

pharmaceutics

Supplementary Materials: Liposome-Embedding Silicon Microparticle for Oxaliplatin Delivery in Tumor Chemotherapy

Armando Cevenini, Christian Celia, Stefania Orrù, Daniela Sarnataro, Maddalena Raia, Valentina Mollo, Marcello Locatelli, Esther Imperlini, Nicoletta Peluso, Rosa Peltrini, Enrica De Rosa, Alessandro Parodi, Luigi Del Vecchio, Luisa Di Marzio, Massimo Fresta, Paolo Antonio Netti, Haifa Shen, Xuewu Liu, Ennio Tasciotti and Francesco Salvatore

S2.1. Stock Solution, Calibration Curve and Quality Control (QC) Analysis

1 mg of oxa and carboplatin (Internal Standard, IS)) were dissolved in MilliQ water (2 mL) to have final stock solutions of 500 µg/mL of standard solutions. Oxa standard solution was further diluted to have working standards in the range of concentration from 5 to 250 µg/mL as previously reported [1]. The working solution were further diluted (1:10 v/v) with methanol thus obtaining working samples for the HPLC analysis in the range from 0.5 to 20 µg/mL. IS was added to each sample before running the HPLC analysis. The matrix-matched calibration standards were obtained by mixing oxa with various liposomal formulations (Tab. 1) and then extracting drug from the resulting mixture before HPLC analysis. The high, medium and low QC values of oxa obtained during the analysis were 15, 7.5, 1.5 µg/mL, respectively.

Liposomes (180 µL) was mixed with the oxa solutions (10 µL) and IS (10 µL, 25 µg/mL), then vortex-mixed for 3 min. The modification of samples in liposomes was: 10% (v/v) for calibration-curve and QC samples, and 5% (v/v) for real samples, respectively. $ZnSO_4$ (50 µL, 5% w/v) was dissolved in a mixture of MiliQ water:methanol:AcN (5:3:2 v/v) and used to precipitate samples. The solution was added to the different samples, which were vortex-mixed (40 Hz) and then centrifuged at 12,000 × g for 10 min at 4°C. Supernatant was withdrawn, filtered through Phenex-PTFE (4 mm, 0.45 µm) syringe filters (Phenomenex, Torrance, CA,USA), and finally transferred in the glass tubes. Samples (20 µL) were finally analyzed with a HPLC system as herein reported in Section 2.8 of Materials and Methods in the main paper.

The analytical method was validated according to International Guidelines [2]. Limit of Detection (LOD), Limit of Quantification (LOQ), linearity, intra- and inter-day trueness and precision, selectivity, recovery, stability and parallelism testing of oxa used during the analysis were measured. Results were an average of three independent experiments and are reported in Table S2.

Table S1. HPLC gradient elution for the analysis of oxa in liposomal formulations.

Time (min)	Flow (ml/min)	A% (v/v)[1]	B% (v/v)[2]
0	1.0	98	2
2	1.0	98	2
4	1.0	70	30
6	1.0	70	30
10	1.0	98	2
10.50	1.0	98	2
18.50	1.0	98	2
0	1.0	100	0

[1]Mobile phase A = MilliQ water; [2]Mobile phase B = Acetonitrile.

Table S2. Recovery values obtained with the weighted-linear least-squares regression analysis of six independent eight non-zero concentration points of oxa with liposomal formulations.

	**Oxaliplatin**	
	Intra-day	Inter-day
QC[1] low standard	1.5 μg/mL	
QC[1] low measured	1.27 μg/mL	1.32 μg/mL
Standard Deviation	0.15	0.03
RSD%	11.83	2.24
Bias%	−15.0	−12.07
QC[1] medium standard	7.5 μg/mL	
QC[1] medium measured	6.10 μg/mL	6.79 μg/mL
Standard Deviation	0.06	0.25
RSD%	1.04	4.11
Bias%	−18.70	−9.50
QC[1] high standard	17 μg/mL	
QC[1] high measured	15.80 μg/mL	15.06 μg/mL
Standard Deviation	0.37	0.71
RSD%	2.42	4.68
Bias%	−7.05	−11.40

[1]QC = quality control of the analysis.

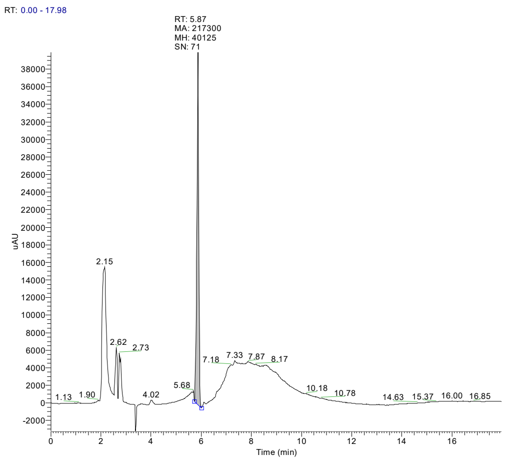

(**A**)

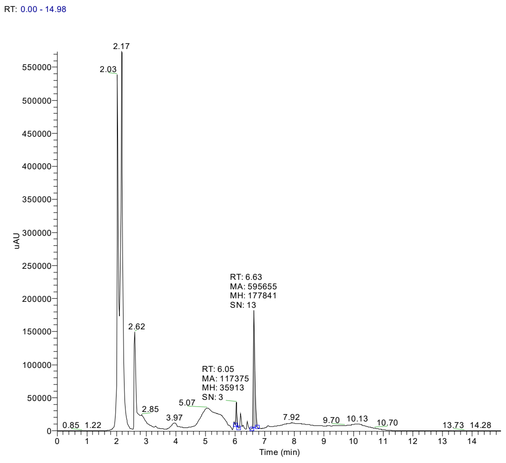

(**B**)

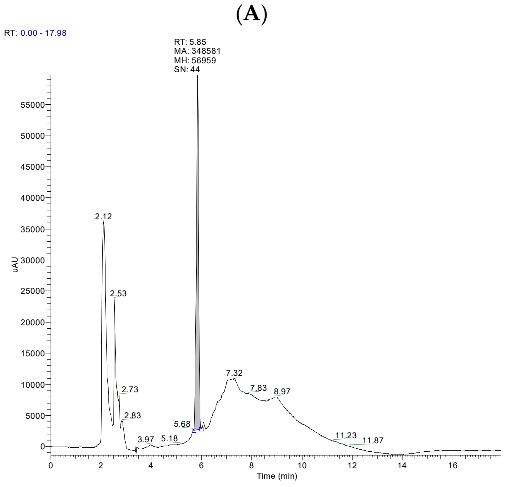

(**C**)

Figure S1. HPLC chromatograms of oxa (**A**), oxa and IS (**B**), and oxa-loaded liposomes (**C**) after separation and quantification by HPLC analysis (section 2.8 of Materials and Methods).

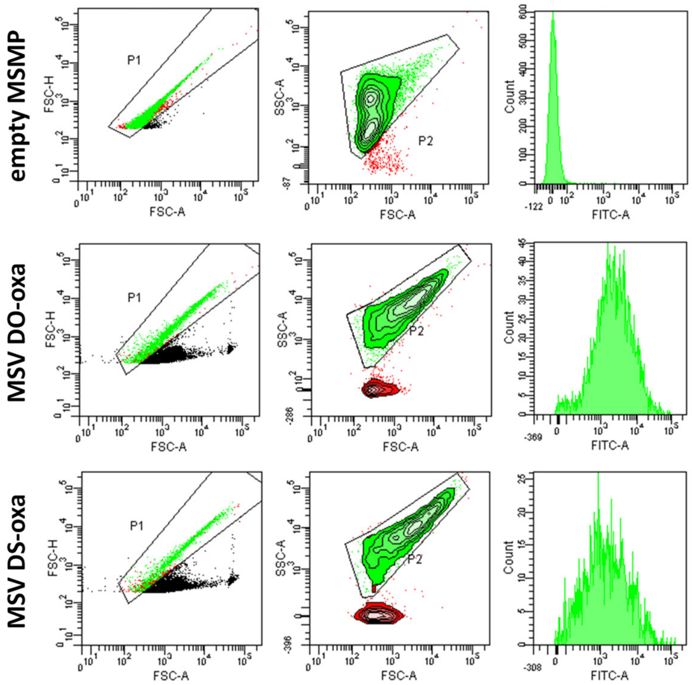

Figure S2. Cytofluorimetryic analysis of multistage vector (MSV) assembly. MSV particles were resuspended in 10 mM Hepes pH 7.4 and analyzed by BD FACSCanto II and BD FACSDiva™ software (BD Biosciences, Franklin Lakes, NJ, USA). The events, properly gated on FSC-A/SSC-A and FSC-H/FSC-A plots, were analyzed for the mean fluorescence intensity (MFI) of the oregon-green-labeled liposomes contained in the MSV. Empty mesoporous silicon microparticles (MSMP) were used as a negative control. The figure is representative of one out of three independent experimental replicates.

References

1. Malatesta, L.; Cosco, D.; Paolino, D.; Cilurzo, F.; Costa, N.; Di Tullio, A.; Fresta, M.; Celia, C.; Di Marzio, L.; Locatelli, M. Simultaneous quantification of Gemcitabine and Irinotecan hydrochloride in rat plasma by using high performance liquid chromatography-diode array detector. *Journal of Pharmaceutical and Biomedical Analysis* **2018**, *159*, 192-199
2. Guideline, ICH Harmonised Tripartite. "Validation of analytical procedures: text and methodology Q2 (R1)." International conference on harmonization, Geneva, Switzerland. Vol. 11. **2005**.
